# Severity of back pain may influence choice and order of practitioner consultations across conventional, allied and complementary health care: a cross-sectional study of 1851 mid-age Australian women

**DOI:** 10.1186/s12891-016-1251-0

**Published:** 2016-09-17

**Authors:** David Sibbritt, Romy Lauche, Tobias Sundberg, Wenbo Peng, Craig Moore, Alex Broom, Emma Kirby, Jon Adams

**Affiliations:** 1 Faculty of Health, University of Technology Sydney, PO Box 123, Ultimo, NSW 2007 Australia; 2 Research Group for Studies of Integrative Health Care, Karolinska Institutet, Solnavägen 1, 171 77 Solna, Sweden; 3 Faculty of Arts and Social Sciences, University of New South Wales, Sydney, NSW 2052 Australia

**Keywords:** Back pain, Health service utilisation, Complementary medicine, Women

## Abstract

**Background:**

Back pain is a common, disabling and costly disorder for which patients often consult with a wide range of health practitioners. Unfortunately, no research to date has directly examined the association between the severity of back pain and back pain sufferers’ choice of whom and in what order to consult different health practitioners.

**Methods:**

This is a sub-study of the large nationally representative Australian Longitudinal Study on Women’s Health (ALSWH). The mid-age cohort women (born 1946-51, *n* = 13,715) of the ALSWH were recruited from the Australian national Medicare database in 1996. These women have been surveyed six time, with survey 6 being conducted in 2010 (*n* = 10,011). Mid-age women (*n* = 1851) who in 2010 had sought help from a health care practitioner for their back pain were mailed a self-report questionnaire targeting their previous 12 months of health services utilisation, health status and their levels of back pain intensity.

**Results:**

A total of 1620 women were deemed eligible and 1310 (80.9 %) returned completed questionnaires. Mid-age women with back pain visited various conventional, allied health and CAM practitioners for care: 75.6 % consulted a CAM practitioner; 58.4 % consulted a medical doctor; and 54.2 % consulted an allied health practitioner. Women with the most severe back pain sought conventional care from a general practitioner, and those who consulted a general practitioner first had more severe back pain than those who consulted another practitioner first. Following the general practitioner visit, the women with more severe back pain were more likely to be referred to a conventional specialist, and those with less severe back pain were more likely to be referred to a physiotherapist.

**Conclusions:**

Our findings suggest that women with more severe back pain are likely to visit a conventional practitioner first, whereas women with less severe back pain are likely to explore a range of treatment options including CAM practitioners. The improvement of back pain over time following the various possible sequencing of consultations with different types of health practitioners is a topic with implications for ensuring safe and effective back pain care and worthy of further detailed investigation.

## Background

Back pain (BP) is pain that targets the posterior aspect of the body in the area of the lumbar spine and pelvis [1]. The global age-standardised point prevalence of BP has been estimated to 9 % and appears to have remained stable over recent decades [2]. BP is a burden not only for the individual sufferer [3] but also for their families, has major cost impacts for health care systems [4] and leads to even greater indirect cost burden through loss of job productivity and inefficiencies [5, 6]. Back pain severity shows substantial influence in individuals’ choice of health care consultations and treatments. A previous randomized controlled trial found only patients with moderate or severe baseline pain intensity was associated with the reductions in mean back pain intensity via taking tapentadol [7]. A longitudinal analysis reported the finding that changes in pain severity predicted subsequent depression severity in primary care [8]. Further, a clinical practice guideline strongly recommended all health care practitioners to assess the severity of baseline pain when diagnosing and treating back pain as the diverse medications used have different benefits for patients with varied severity of back pain [9].

BP has long been reported as a highly prevalent complaint in Australian primary care [10] and a multitude of health care provider options exist for back pain - the use of which in many cases attract out-of-pocket spending by consumers in Australia [5]. BP care options include a range of conventional medicine providers (e.g. specialists), allied health providers (e.g. physiotherapists, pharmacists) and complementary and alternative medicine (CAM) providers (e.g. massage therapists, chiropractors, osteopaths) [11]. In Australia, primary health care is typically a person's first point of contact with the health system and is most often provided outside the hospital system. A person does not need a referral for this level of care, which includes services provided by general practitioners, allied health professionals and CAM practitioners. Primary health care is part of a larger system involving other services and sectors, and so can be considered as the gateway to the wider health system. Through assessment and referral, individuals are directed from one primary care service to another, and from primary services onto secondary specialist health care (which is facilitated by the GP) or onto other health services, and back again. Secondary health care is medical care provided by a specialist or facility upon referral by a primary care physician. It includes services provided by hospitals and specialist medical practices [12].

While population-based research in Australia has reported that significant numbers of back pain sufferers choose not to seek care [11, 13, 14], those that do seek care are more likely to be females and with more severe pain and disability [14]; visiting both conventional and CAM practitioners [15], in particular general practitioners and chiropractors [14] as well as massage therapists [13]. It is common for people in mid-age to experience back pain [16]. In addition, mid-age women are more likely to utilise a range of health care practitioners for the treatment of chronic illness, including back pain [15]. Furthermore, recent research also shows a very small percentage of BP sufferers seek exclusive help from CAM and over 50 % seek help from only conventional providers [13]. It is interesting to note the varied characteristics of patients with back pain who sought help in general practice and chiropractic practice. Patients with back pain treated by general practitioners were younger, more likely to be a smoker, and experienced greater pain than those treated by chiropractors [17–19], and the back pain sufferers’ self-referral to chiropractors was likely associated with acute back pain, while self-referral to general practitioners was likely associated with chronic back pain [18].

To date, there are variable levels of evidence showing improved patient outcomes for low back pain available via a range of different treatment approaches and providers [20–22]. How to provide care in a way that reflects best clinical practice across a range of individual circumstances and variables is reflected in this wide range of clinical approaches [23, 24]. Recent work examining women's practitioner choices for BP care suggests that choice of treatment is largely unrelated to the relative evidence-base of those treatments, being instead influenced by the patient’s experience and familiarity of care, wider social network recommendations and the geographical proximity of seemingly qualified practitioners [25]. Indeed, it has also been suggested that the range of BP management options available to sufferers may lead to increased but not necessarily more effective BP health care use constituting a challenge to providing cost-efficient care and health services for BP [26].

Despite these complexities around the wide range of BP care options available, no research to date has examined the association between the severity of BP or general health and BP sufferers’ choice of whom and in what order to consult different health practitioners. In response, this paper reports the first such examination amongst a nationally representative sample of mid-age women with BP.

## Methods

### Sample

This research was conducted as part of the Australian Longitudinal Study on Women’s Health (ALSWH), which was designed to investigate multiple factors affecting the health and well-being of women in Australia over a 20-year period. Women in three age groups (“young” 18–23, “mid age” 45–50 and “older” 70–75 years) were randomly selected from the national Medicare database, which is maintained by the Australian Government and covers all Australian citizens and permanent residents’ name and address details [27]. The age groups for the main cohorts were selected so that participation would commence, at least for most women in the cohorts, before the occurrence of major life events, such as first pregnancy, establishment of a long-term relationship, menopause, retirement, or death of a partner [27]. The focus of this study is women from the “mid-age” cohort. The baseline survey (*n* = 14,779) was conducted in 1996 and the respondents have been shown to be broadly representative of the national population of women in the target age group [28]. Socio-demographic characteristics, health services, health behaviours and risk factors, physical health, emotional health, and time use were collected from the survey questionnaires. For this cross-sectional sub-study, 1851 women who had indicated in survey 6 (conducted in 2010) that they had sought help from a health care practitioner for their back pain were mailed an invitation to participate and a questionnaire. Of these women, 1620 were deemed eligible, and 1310 (80.9 %) returned completed sub-study questionnaires. At the time of this survey, the women were aged 59–64 years.

### Health service utilisation - outcome measures

The women were asked if they had consulted with a range of commonly consulted medical doctors, a range of allied health practitioners and a range of CAM practitioners for their back pain in the previous 12 months. The list of medical doctors included: general practitioner (GP), orthopaedic specialist, neurologist, rheumatologist, or other medical practitioner. The list of allied health practitioners included: physiotherapist, occupational therapist, nurse, pharmacist, or other allied health practitioner. The list of CAM practitioners included: massage therapist, chiropractor, herbalist/naturopath, meditation/yoga therapist, acupuncturist, osteopath, reflexologist, traditional Chinese medicine therapist, aromatherapist, craniosacral therapist, reiki therapist, or other CAM practitioner. In addition to asking which health care practitioners the women consulted for their back pain, the women were also asked in which order they consulted each health care practitioner for their back pain.

### Health status

The women were asked to indicate how frequently (‘never’, ‘rarely’, ‘intermittently’, ‘regularly’, and ‘continuously’) they experienced the back pain in the previous 12 months, which was categorised as continuous back pain or not continuous back pain. The women were also asked about their *typical* level of back pain intensity and their *worst* level of back pain intensity over the previous 12 months on a scale from 0 to 10, with 0 representing ‘no pain’ and 10 representing ‘worst possible pain’. It has previously been shown that it is valid to use a 12 month recall period using such a scale [29, 30]. The Short-Form 36 (SF-36) Quality of Life questionnaire was used to produce a measure of health status and quality of life, with higher scores representing better health. Results of the SF-36 were transformed into eight mental and physical health subscales [31]; all subscales were considered for analyses.

### Statistical analyses

Descriptive statistics were used to assess the frequency and percentages of all the included medical doctors, allied health practitioners, and alternative health practitioners. Comparisons were made between women consulting a GP first, women consulting chiropractor first, women consulting physiotherapist first, women consulting massage therapist first, and women consulting other therapist first regarding the characteristics of their back pain (back pain frequency, typical back pain intensity, worst back pain intensity) and their quality of life (all eight domains of SF-36). Comparisons between two categorical variables were made using the chi-square test. Comparisons between continuous and categorical variables were made using analysis of variance (ANOVA). To correct for multiple comparisons, a modified Bonferroni test was used [32]. At most, there was minimal missing data for any variable (the maximum was 2 % missing for SF36 General Health) and as such, data were analysed *as is*. All analyses were conducted using the statistical software Stata, version 11. *P*-values less than 0.05 were considered statistically significant.

## Results

The main questionnaire item used in the analyses was the order in which the women consulted each health care practitioner for their back pain. There were 116 women who were excluded from the analyses due to inconsistencies in their response to this question (e.g. they ticked more than one health care practitioner as being the first practitioner they consulted), leaving a total sample size of 1194.

The frequency of consultations with a medical doctor, allied health practitioner and/or CAM practitioner in the previous 12 months for back pain is presented in Table 1. On average, the women consulted 3.0 (95 % CI: 2.8, 3.1) health care practitioners over a 12 month period, for their back pain. In total, 697 (58.4 %) women consulted a medical doctor for their back pain. General practitioners (*n* = 664, 55.6 %) were the most commonly consulted medical doctors, followed by orthopaedic specialists (*n* = 94, 7.9 %) and rheumatologists (*n* = 75, 6.3 %). In total, 647 (54.2 %) women consulted an allied health practitioner for their back pain. Physiotherapists (*n* = 430, 36.0 %) were the most commonly consulted allied health practitioner, followed by pharmacists (*n* = 243, 20.4 %) and nurses (*n* = 40, 3.4 %). In total, 903 (75.6 %) women consulted a CAM practitioner for their back pain. Massage therapists (*n* = 515, 43.1 %) were the most commonly consulted CAM practitioner, followed by chiropractors (*n* = 441, 36.9 %) and acupuncturists (*n* = 154, 12.0 %).

**Table 1:** Frequency of consultations with doctors, allied health practitioners and/or alternative practitioners for back pain

Health care practitioner	Frequency	Percent
Medical doctors		
General practitioner	664	55.6
Orthopaedic specialist	94	7.9
Rheumatologist	75	6.3
Neurologist	47	3.9
Other medical practitioner	42	3.5
*Any medical doctor*	*697*	*58.4*
Allied health practitioners		
Physiotherapist	430	36.0
Pharmacist	243	20.4
Nurse	40	3.4
Occupational therapist	28	2.4
Other allied health practitioner	104	8.7
*Any allied health practitioner*	*647*	*54.2*
Alternative health practitioners		
Massage therapist	515	43.1
Chiropractor	441	36.9
Acupuncturist	154	12.9
Herbalist/naturopath	110	9.2
Meditation/yoga therapist	106	8.9
Osteopath	101	8.5
Reiki therapist	37	3.1
Reflexologist	35	2.9
Traditional Chinese medicine therapist	30	2.5
Aromatherapist	23	1.9
Craniosacral therapist	17	1.4
Other alternative health practitioner	196	16.4
*Any alternative health practitioner*	*903*	*75.6*

For approximately half of the women (*n* = 594; 49.7 %) a GP was the first practitioner consulted for their back pain. A chiropractor was the first practitioner consulted for back pain by 20.1 % (*n* = 240) of the women. This is in contrast to the higher percentage of women consulting an allied health practitioner (54.2 %) or a massage therapist (43.1 %) more generally for back pain in the previous 12 months, while a physiotherapist and a massage therapist were the first practitioners consulted by 7.2 % (*n* = 86) and 4.6 % (*n* = 55) of the women respectively. Of the remaining women, 7.9 % (*n* = 119) consulted an ‘other’ practitioner first for their back pain and 10.5 % did not consult a practitioner for their back pain (Table 2).

**Table 2:** Comparisons between the health care practitioner first consulted for back pain by women across measures of back pain frequency, back pain intensity and SF-36 dimensions of quality of life

Characteristics	Health care practitioner first consulted for back pain*					
	General practitioner	Chiropractor	Physiotherapist	Massage	Other	*p*-value***
	(*n* = 594)	(*n* = 240)	(*n* = 86)	(*n* = 55)	(*n* = 119)	
	% (95 % C.I.)	% (95 % C.I.)	% (95 % C.I.)	% (95 % C.I.)	% (95 % C.I.)	
Back pain frequency						
Continuous back pain (% yes)^a, b, c, d^	22 (19, 26)	7 (4, 10)	11 (4, 17)	9 (1, 17)	11 (5, 17)	<0.001
	Mean (95 % C.I.)	Mean (95 % C.I.)	Mean (95 % C.I.)	Mean (95 % C.I.)	Mean (95 % C.I.)	
Back pain intensity**						
Intensity of *typical* back pain^a, b, c, d^	5.8 (5.6, 5.9)	4.7 (4.4, 4.9)	4.6 (4.2, 5.0)	4.4 (3.9, 4.9)	5.2 (4.9 5.5)	<0.001
Intensity of *worst* back pain^a, b, c^	7.7 (7.5, 7.8)	6.8 (6.5, 7.0)	6.6 (6.0, 7.1)	6.5 (5.9, 7.1)	7.2 (6.8, 7.6)	<0.001
SF-36 dimensions						
General health^a, b, c, d^	56.9 (55.1, 58.7)	67.5 (64.8, 70.1)	69.4 (65.4, 73.5)	70.7 (65.3, 76.1)	63.7 (59.8, 67.6)	<0.001
Bodily pain^a, b, c, d^	45.1 (43.4, 46.8)	57.4 (54.7, 60.2)	56.1 (51.8, 60.5)	61.3 (55.3, 67.3)	52.6 (48.8, 56.3)	<0.001
Physical functioning^a, b, c, d^	59.3 (57.2, 61.3)	72.7 (69.7, 75.6)	71.9 (67.2, 76.6)	78.2 (73.5, 82.8)	68.5 (64.3, 72.7)	<0.001
Role physical^a, b, c^	42.2 (38.8, 45.5)	62.3 (56.9, 67.7)	63.4 (54.5, 72.2)	69.1 (58.5, 79.7)	53.6 (45.6, 61.6)	<0.001
Mental health^a, c^	70.3 (68.8, 71.9)	74.9 (72.7, 77.1)	74.8 (71.3, 78.4)	77.0 (72.9, 81.1)	73.7 (70.5, 76.9)	0.002
Role emotional^a, b, c^	68.0 (64.6, 71.3)	79.0 (74.4, 83.6)	84.1 (77.3, 90.9)	93.3 (87.9, 98.8)	76.6 (69.6, 83.6)	<0.001
Vitality^a, b^	48.0 (46.3, 49.8)	55.5 (52.9, 58.2)	57.6 (53.5, 61.7)	55.8 (50.0, 61.5)	52.2 (48.1, 56.4)	<0.001
Social functioning^a, b, c^	69.1 (66.8, 71.3)	80.3 (77.2, 83.5)	84.3 (80.0, 88.6)	81.1 (75.1, 87.2)	75.4 (70.5, 80.4)	<0.001

*Note that 100 women did not consult any health care practitioner for their back pain within the previous 12 months

**Back Pain Intensity, as measured on a scale from 0 to 10, with 0 representing ‘no pain’ and 10 representing ‘worst possible pain’

***p-value was determined by chi-square test or analysis of variance (ANOVA), where appropriate

^a^Statistically significant difference between GP and chiropractor, Bonferroni adjustment (α = 0.05)

^b^Statistically significant difference between GP and physiotherapist, Bonferroni adjustment (α = 0.05)

^c^Statistically significant difference between GP and massage therapist, Bonferroni adjustment (α = 0.05)

^d^Statistically significant difference between GP and other, Bonferroni adjustment (α = 0.05)

Table 2 shows comparisons made between the women based on the health care practitioner consulted first (i.e. GP consulted first, chiropractor consulted first, physiotherapist consulted first, massage therapist consulted first, and ‘other’ health care practitioner consulted first) across a number of measures including frequency of back pain, intensity of back pain, as well as SF-36 quality of life dimensions, respectively. In terms of back pain frequency, 22 % of the women who consulted a GP first experienced back pain continuously beforehand, compared to only 7–11 % of women who consulted any of the other practitioners first (*p* < 0.001). Similarly, women who consulted a GP first experienced more intense *typical* and *worst* back pain than women who consulted any of the other practitioners first (both *p* < 0.001). Women who consulted a GP first also had significantly lower levels of general health, bodily pain, physical functioning, role physical, role emotional, vitality, social functioning (SF-36) than women who consulted any of the other practitioners first (all *p* < 0.001) and lower levels of mental health (SF-36) than those women who consulted any other practitioner first (*p* = 0.002).

**Fig. 1:**
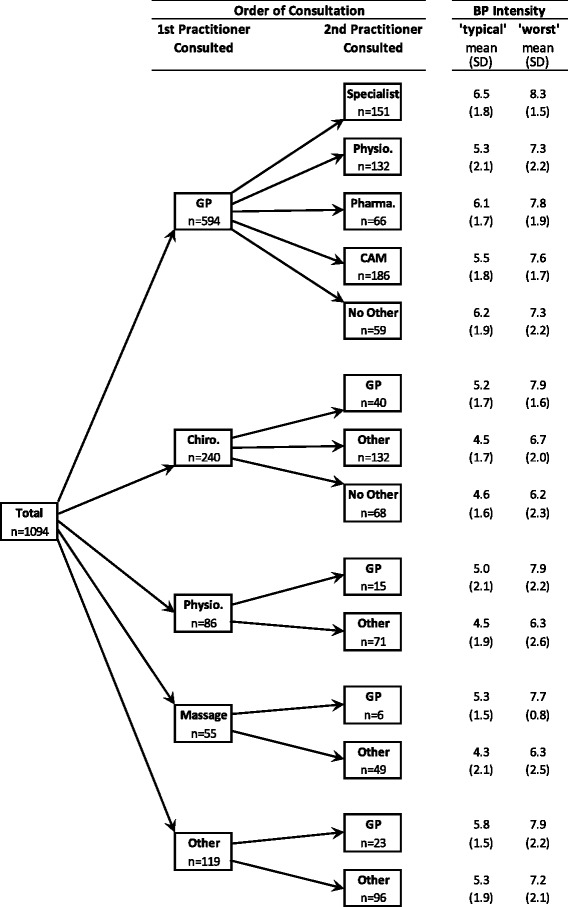
Partitioning of the sample based on order of consultation with any healthcare practitioners*. *Back pain (BP) intensity = mean back pain intensity score, as measured on a scale from 0 to 10, with 0 representing ‘no pain’ and 10 representing ‘worst possible pain’

Figure 1 presents a partitioning of the sample based on the order of consultation with any health care practitioner by women for their BP, with associated mean BP intensity scores. For the first practitioner consulted, the sample was partitioned into the 4 most commonly consulted practitioner groups as well as an ‘other’ category (referring to the wide range of health care practitioners excluding GPs, chiropractors, physiotherapists, massage therapists). For the second practitioner consulted, the commonly consulted practitioner groups included GPs, specialists (e.g. orthopaedic specialists, neurologists, and rheumatologists), physiotherapists, pharmacists, CAM practitioners, ‘other’ (referring to any other health care practitioners), and ‘no other’ (referring to the solo consultation with the first practitioner group). In the partitioning of the GP group, it can be seen that for those women who next consulted a specialist (after consulting a GP first) their ‘typical’ and ‘worst’ BP intensity levels were higher than the women who next consulted a physiotherapist (*p* < 0.05), pharmacist, CAM practitioner (*p* < 0.05) or ‘no other’ practitioner. Conversely, for those women who next consulted a physiotherapist (after consulting a GP first) their ‘typical’ and ‘worst’ BP intensity levels were lower than women who next consulted a specialist (*p* < 0.05), pharmacist, CAM practitioner, or ‘no other’ practitioner (*p* < 0.05). In the partitioning of the chiropractic group, it can be seen that for those women who next consulted a GP (after consulting a chiropractor first) their ‘typical’ and ‘worst’ BP intensity levels were higher than the women who next consulted a ‘other’ practitioner or ‘no other’ practitioner, which was statistically significant for the ‘worst’ BP intensity levels (*p* < 0.05). In the partitioning of the physiotherapist, massage therapist and ‘other’ groups, there was a common pattern in that for those women who next consulted a GP (after consulting either a physiotherapist, massage therapist or ‘other’ practitioner first) their ‘typical’ and ‘worst’ BP intensity levels were higher than the women who consulted an ‘other’ practitioner, although none of these differences were statistically significant. It is interesting to note that all participants who first consulted a physiotherapist, massage therapist or ‘other’ saw an additional provider afterwards.

## Discussion

This study of a large, nationally-representative sample of Australian women aged 59–64 years with BP provides the first examination of the severity of BP and its association to BP sufferers’ choice of whom and in which order to consult a range of conventional, allied health and CAM providers. The study presents 3 key findings related to: whom BP sufferers’ seek help from; the sequencing of practitioner use from whom these BP sufferers seek help; and the association between severity of BP and BP sufferers’ choice of which practitioner from whom to seek help.

First, the study shows that mid-age female BP sufferers consult with a wide range of conventional, allied health and CAM practitioners in response to their BP. In line with previous research examining health care utilisation for BP [11, 13, 15, 33], our analyses illustrate that a substantial percentage of women with BP do not exclusively consult a conventional, allied or CAM provider for their BP but instead utilise the services of different practitioner types sequentially over their patient journey. This finding suggests these women may be adopting a pragmatic approach to pain management free from an allegiance or preconception related to any specific provider or approach to treatment [25].

Our analyses also provide the first focused examination of the sequencing of practitioner use amongst BP sufferers as associated with BP severity. Interestingly, the study shows women with the most severe BP (either initially or ultimately) are significantly more likely to seek care from a GP than from any other practitioner group. While our analyses do not accommodate the opportunity to investigate and explain this finding, there are at least three prominent possibilities. This finding may be due to women possibly having a pre-established long-term relationship with their GP and that as such they seek consultation with their GP as a first port-of-call and gatekeeper/advisor to other services (for BP as for other conditions) [34]. On the other hand, this finding may be due to women’s own perceptions of the severity of their BP. It may be these women feel more comfortable consulting a GP when they perceive their BP to be (unusually) severe and where they perceive the need for a more trusted authority and/or a provider who has greater access to more advanced diagnostic investigations (eg. MRI, CT, blood tests) [35]. A third possibility is that some women in more severe pain may be aware of the ‘faster relief’ that is more likely available through prescriptive medicine [36] or that the conventional medical approach may require less personal time and effort compared to the more active-care model typically provided through a physiotherapy or a chiropractic. Another possible explanation for this finding may be related to back pain sufferers’ self-payment. The out-of-pocket expense of the GP consultations is lower than that of the CAM practitioner consultations and that of the allied health practitioner consultations amongst women with back pain [3]. Ultimately, further research is needed to fully examine and help understand the detailed decision-making of women with BP regarding the association between the severity of their BP and their choice of who to initially consult.

Focusing upon just those women who consult with a GP first for their BP, our study also identified those women with more severe BP as more likely to then subsequently consult with a specialist. While, in contrast, those women who consult with a GP first but who report less severe BP were more likely to next consult with a physiotherapist or CAM practitioner. The choice who to consult subsequent to GP consultation may be influenced by several factors. On the one hand, GPs might be more likely to refer more severely affected patients to a specialist as this might indicate a more serious cause of BP, especially when the patient also presents with concurrent red-flag findings that may require more advanced clinical investigation [37]. GPs may also believe physical therapy to be less effective for some BP categories, such as acute and sub-acute low back pain, where clinical evidence is still poorly validated compared to chronic low back pain [38, 39]. On the other hand, patients who consult their GP may also have a strong preference towards subsequent therapies; and those more severely affected might demand to see a specialist rather than any other health care provider. It is also unclear how much influence the lower levels of physical function and mental health found more often in women who first present to GPs may have on the selection of follow-up care. That women may be presenting to GPs with these additional health factors may further influence routine GP clinical decision-making and referral patterns, especially for those who do not otherwise present with red-flag findings that would otherwise lead to a specialist referral [40]. Since our analyses cannot provide information about whether the second practitioner was consulted due to referral or choice, these assumptions remain speculative and require further detailed empirical investigation.

Regarding those women with BP who consulted a practitioner other than a GP first, our results indicate that approximately 1 in 5 later consult a GP and that these particular women report more severe BP than those not consulting their GP as either first or second choice of provider. It would appear that while women with BP may initially consult from a wide range of provider types, those women with more severe BP will eventually return to or initiate GP care. Our study does not accommodate direct examination of the reasons for this specific consultation pattern, but it appears that in these cases the consultation with an allied or CAM practitioner may have failed to deliver satisfactory pain relief. For example, this outcome may more likely occur for those types of low back pain that are currently less proven to be responsive to physical therapies (acute and sub-acute verses chronic low back pain) or where lower back pain is further complicated by potentially more serious medical red-flag clinical findings (spinal stenosis, disc prolapse, spinal instability) [41, 42]. What does require further investigation is whether the return to or initiation of GP care at this later stage in the patient journey is primarily led by the patient (with or without the knowledge and/or support of the practitioner currently being consulted) or the current health care practitioner, and to examine the extent to which others (family/friends and informal carers/support people) may influence this aspect of the women’s decision-making.

One limitation of our study is that BP and health care utilisation are self-reported, potentially introducing a recall bias. Additionally, BP status was defined in our study by the self-reporting of a single question. This lack of confirmatory diagnosis could potentially bias the findings. However, existing research has evidenced the validity, and comparability to medical record assessments, of a questionnaire-based measure of comorbidity [43] and further, these limitations are offset by the opportunity to analyse data from a large nationally-representative sample of mid-age women with BP. Another limitation to this study is the fact that pain level is only one variable that may influence patient and provider decision-making. There are other factors that may also influence decision-making about care either in association with pain levels or separate. For example, patient decision-making may be further influenced by the increasing comparative costs associated with some care options, especially toward treatment available outside of government funded medical care. Provider decision-making may be further influenced by patterns of referral associated with their knowledge or awareness of the evidence to support some treatment options or concerns about adverse treatment reactions or patient findings outside of pain levels that may require further investigation.

## Conclusions

Our findings suggest that women with more severe back pain are likely to visit a conventional medical practitioner first, whereas women with less severe back pain are more likely to explore a range of provider options including CAM practitioners. Both the detailed reasons for such provider use as well as the improvement of back pain over time following the various possible sequencing of consultations with different types of health practitioners is a topic with implications for ensuring safe and effective back pain care and worthy of further detailed investigation.

## Abbreviations

ALSWH: Australian longitudinal study on women’s health
ANOVA: Analysis of variance
BP: Back pain
CAM: Complementary and alternative medicine
GP: General practitioner
MH: Mental health
PF: Physical function
